# Cor pulmonale: the role of traditional and advanced echocardiography in the acute and chronic settings

**DOI:** 10.1007/s10741-020-10014-4

**Published:** 2020-08-29

**Authors:** Giulia Elena Mandoli, Carlotta Sciaccaluga, Francesco Bandera, Paolo Cameli, Roberta Esposito, Antonello D’Andrea, Vincenzo Evola, Regina Sorrentino, Alessandro Malagoli, Nicolò Sisti, Dan Nistor, Ciro Santoro, Elena Bargagli, Sergio Mondillo, Maurizio Galderisi, Matteo Cameli, on behalf of Working group of Echocardiography of Italian Society of Cardiology (SIC)

**Affiliations:** 1 Department of Medical Biotechnologies, Division of Cardiology, AOUS Policlinico Santa Maria alle Scotte, University of Siena, Viale Bracci 1, 53100 Siena, Italy; 2 Cardiology University Department, Heart Failure Unit, IRCCS, Policlinico San Donato, San Donato Milanese and Department of Biomedical Sciences for Health, University of Milano, Milan, Italy; 3 Respiratory Diseases Unit, Department of Medical and Surgical Sciences and Neuroscience, University of Siena, Siena, Italy; 4 Department of Advanced Biomedical Science, Federico II University Hospital Naples, Naples, Italy; 5 Cardiology Department, Echocardiography Lab and Rehabilitation Unit, Monaldi Hospital, Second University of Naples, Naples, Italy; 6 Department of Health Promotion Sciences, Maternal-Infant Care, Internal Medicine and Specialities of Excellence “G. D’Alessandro”, University of Palermo, Cardiology Unit, University Hospital P. Giaccone, Palermo, Italy; 7 Division of Cardiology, Nephro-Cardiovascular Department, “S. Agostino-Estense” Public Hospital, University of Modena and Reggio Emilia, Modena, Italy; 8 Institute for Emergency Cardiovascular Diseases and Transplant Targu Mures, Targu Mures, Romania

**Keywords:** Cor pulmonale, Pulmonary hypertension, Right heart failure, Echocardiography, Right ventricular dysfunction

## Abstract

Cor pulmonale is the condition in which the right ventricle undergoes morphological and/or functional changes due to diseases that affect the lungs, the pulmonary circulation, or the breathing process. Depending on the speed of onset of the pathological condition and subsequent effects on the right ventricle, it is possible to distinguish the acute cor pulmonale from the chronic type of disease. Echocardiography plays a central role in the diagnostic and therapeutic work-up of these patients, because of its non-invasive nature and wide accessibility, providing its greatest usefulness in the acute setting. It also represents a valuable tool for tracking right ventricular function in patients with cor pulmonale, assessing its stability, deterioration, or improvement during follow-up. In fact, not only it provides parameters with prognostic value, but also it can be used to assess the efficacy of treatment. This review attempts to provide the current standards of an echocardiographic evaluation in both acute and chronic cor pulmonale, focusing also on the findings present in the most common pathologies causing this condition.

## Background

**Fig. 1.**
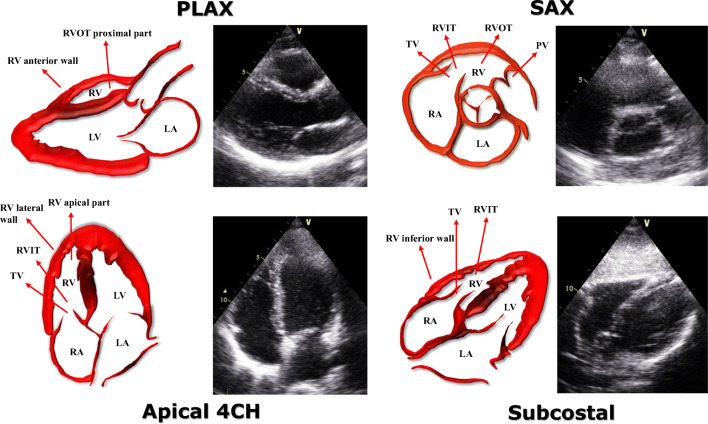
Echocardiographic assessment of the right ventricle. This figure shows the main four 2D-echocardiographic views that should be assessed for a thorough evaluation of the right ventricle (RV): parasternal long axis view (PLAX), short axis view (SAX), apical 4-chamber view, and subcostal view. CH, chambers; LA, left atrium; LV, left ventricle; PLAX, parasternal long-axis view; PV, pulmonary valve; RA, right atrium; RV, right ventricle; RVIT, right ventricular inflow tract; RVOT, right ventricular outflow tract; SAX, short axis; TV, tricuspid valve

Cor pulmonale can be defined as the clinical setting in which the right side of the heart, in particular the right ventricle (RV), is affected by a pressure overload that induces changes of RV function and morphology. Depending on the duration of time in which the increased RV afterload is established, it is possible to distinguish an acute cor pulmonale from a chronic form of the disease. The most common condition responsible for the acute type is acute pulmonary embolism (APE), whereas the chronic cor pulmonale is usually caused by chronic obstructive pulmonary disease (COPD) [1], followed by idiopathic pulmonary fibrosis (IPF) and chronic thromboembolic pulmonary hypertension (CTEPH). Commonly, these diseases induce a chronic hypoxemia and/or a remodelling of the pulmonary circulation [2], which forces the RV to adapt in compensation for the increased mechanical work required to pump blood through the lungs. In this regard, the echocardiographic evaluation is a cornerstone in both the diagnosis and the prognostic stratification of these patients. In general, when RV afterload is acutely increased, the results are a dilatation and an impaired function, whereas when the pressure increase is gradual, the RV has time to adapt and is more likely to present complex remodelling features, including RV hypertrophy. Figure 1 outlines the 2D-echocardiographic views that should be always assessed in order to pursue a thorough evaluation of the RV. This review attempts to present the current evidences of the role of echocardiography in both acute and chronic cor pulmonale, including new techniques, such as 3-dimensional echocardiography (3DE) and speckle tracking echocardiography (STE), which have proven valuable tools for distinguishing between the acute and the chronic form.

## Acute cor pulmonale

Acute cor pulmonale might be described as the clinical setting in which the RV afterload rapidly increases, causing dilatation and/or impairment of RV function. In clinical practice, the most common condition responsible for this scenario is APE where an acute obstruction of 30–50% of the cross-sectional area of the pulmonary arterial bed, together with hypoxia-induced vasoconstriction, is responsible for the abrupt increase of pulmonary vascular resistance (PVR), which greatly affects RV function. Table 1 shows reference values of the parameters that should be assessed in a complete echocardiographic exam focused on RV size and function.

**Table 1.** Reference values for right ventricular and right atrial size and function

	Parameter	Normal value range
Right ventricle	RV wall thickness (mm)	1–5
	RV basal diameter (mm)	25–41
	RV mid-cavity diameter (mm)	19–35
	RV longitudinal diameter (mm)	59–83
	RVOT PLAX diameter (mm)	20–30
	RVOT proximal diameter (mm)	21–35
	RVOT distal diameter (mm)	17–27
	TAPSE (mm)	≥ 17
	Pulsed Doppler S wave (cm/s)	≥ 9.5
	RVFAC (%)	≥ 35
	RV 3D EF (%)	≥ 45
	Trans-tricuspid E/A	0.8–2.0
	E-wave deceleration time (ms)	119–224
	fw-RV strain (%)	≤− 20
	Pulmonary acceleration time (ms)	≥ 120
Right atrium	RA area (cm^2^)	< 18
	RA volume (mL/m^2^)	25 ± 7 (men) 21 ± 6 (women)
	RA strain (%)	49 ± 13

3D, 3-dimensional; fw, free wall; EF, ejection fraction; PLAX, parasternal long axis; PSAX, parasternal short axis; RA, right atrium; RV, right ventricle; RVFAC, right ventricular fractional area change; RVOT, right ventricular outflow tract; TAPSE, tricuspid annular plane systolic excursion [3]

According to the latest European Society of Cardiology Guidelines for the diagnosis and management of APE [4], echocardiography plays a key role in the evaluation of patients presenting with hemodynamic instability and suspicion of APE. In this regard, if a computed tomography pulmonary angiography examination is not immediately available, echocardiography must be performed in these patients, in order first to exclude other conditions that might be responsible for shock and second to demonstrate RV dilatation/dysfunction indicating a pressure overload, with emergency reperfusion therapy being justified solely on these criteria [4]. Moreover, performing echocardiography in addition to other testing has been associated with a lower in-hospital mortality in APE, likely due to the fact that it speeds up the diagnostic and therapeutic work-up, especially in critical patients [5, 6]. Furthermore, a recent meta-analysis confirmed the high specificity and low sensitivity of this technique for diagnosis, which makes it a useful tool to rule-in APE, especially at the bedside [7].

Besides the direct assessment of specific signs of embolism, such as mobile thrombi in the RV or right atrium (RA), which occurs in approximately 4% of patients [8], there are several indirect echocardiographic parameters of RV dysfunction that suggest the diagnosis of APE, which are summarized in Table 2.

**Table 2.** Standard indirect echocardiographic signs of acute cor pulmonale due to acute pulmonary embolism

Right ventricle	RV dilatation (increased RV diameters) Increased RV to LV diameter ratio (RV/LV > 0.7 in PLAX or > 1 in apical four-chamber view) Reduced TAPSE Reduced RVFAC Reduced tricuspid E/A 60/60 sign (TRPG < 60 mmHg or TR maximum velocity < 3.9 m/s and pulmonary acceleration time < 60 ms) McConnell’s sign (hypo-akinesia of the RV mid-free wall with normal motion of the apex)
Left ventricle	Abnormal or paradoxical septal motion D-shape of the LV in PSAX
Right atrium	RA dilatation Interatrial septum shift towards the left atrium IVC dilatation (transversal diameter > 2.1 cm), with reduced inspiratory collapse

IVC, inferior vena cava; LV, left ventricle; PLAX, parasternal long axis; PSAX, parasternal short axis; RA, right atrium; RV, right ventricle; RVFAC, right ventricular fractional area change; TAPSE, tricuspid annular plane systolic excursion; TR, tricuspid regurgitation; TRPG, tricuspid regurgitation pressure gradient [9]

*RV dilatation* is one of the main indirect findings in APE (see Table 1 for reference values). Albeit not specific, it has been proven to occur in the presence of RV pressure overload, generally indicates a higher clot burden, and is usually associated with other advanced signs of RV systolic dysfunction [10, 11]. Like RV dilatation, other functional parameters have been correlated to clot burden, as shown in the study conducted by Rodrigues et al. that found an inverse correlation of *Right ventricular fractional area change* (RVFAC) with the severity of the pulmonary vascular bed occlusion [12].

However, some standard echocardiographic parameters of RV longitudinal function, like *TAPSE* and *tricuspid s’ velocity* have shown an inferior sensitivity and specificity and are sometimes normal in acute cor pulmonale. In fact, their reduction might be present only in cases with significant hemodynamic impairment [13], and, as is the case with RV dilatation, generally presenting normal values in patients with a lower clot burden. Conversely, TAPSE can be decreased even when RV systolic pressure is within the normal range [14], while s′ velocity is generally more specific, with values below the lower limit present only when RV pressure is increased [15]. This finding reflects the fact that tricuspid annulus velocity by TDI correlates with both RV relaxation [16] and RV filling pressures [17]. The acute increase of RV afterload induces an elevated wall tension, which might impair both the diastolic and systolic functions of the RV [18].

*Trans-tricuspid E/A ratio* is significantly reduced in the presence of increased RV pressure, and it generally presents a gradual return to normal values once the obstruction is resolved, likewise RVFAC and TAPSE, even though the latter has been shown not to completely recover during follow-up [14].

Additionally, *tricuspid e′ velocity* is impaired even in the presence of normal RV systolic pressures, tricuspid s′ velocities and RV filling pressures, which might highlight its role as an early marker of RV dysfunction [15].

Besides its diagnostic role, RV dilatation and dysfunction, identified by echo in hemodynamically stable APE patients, play a key role in disease severity evaluation and risk stratification, in addition to laboratory markers of myocardial damage [4]. Also, follow-up echocardiographic evaluations can aid clinical management and improve outcome prediction in these patients [19].

**Fig. 2.**
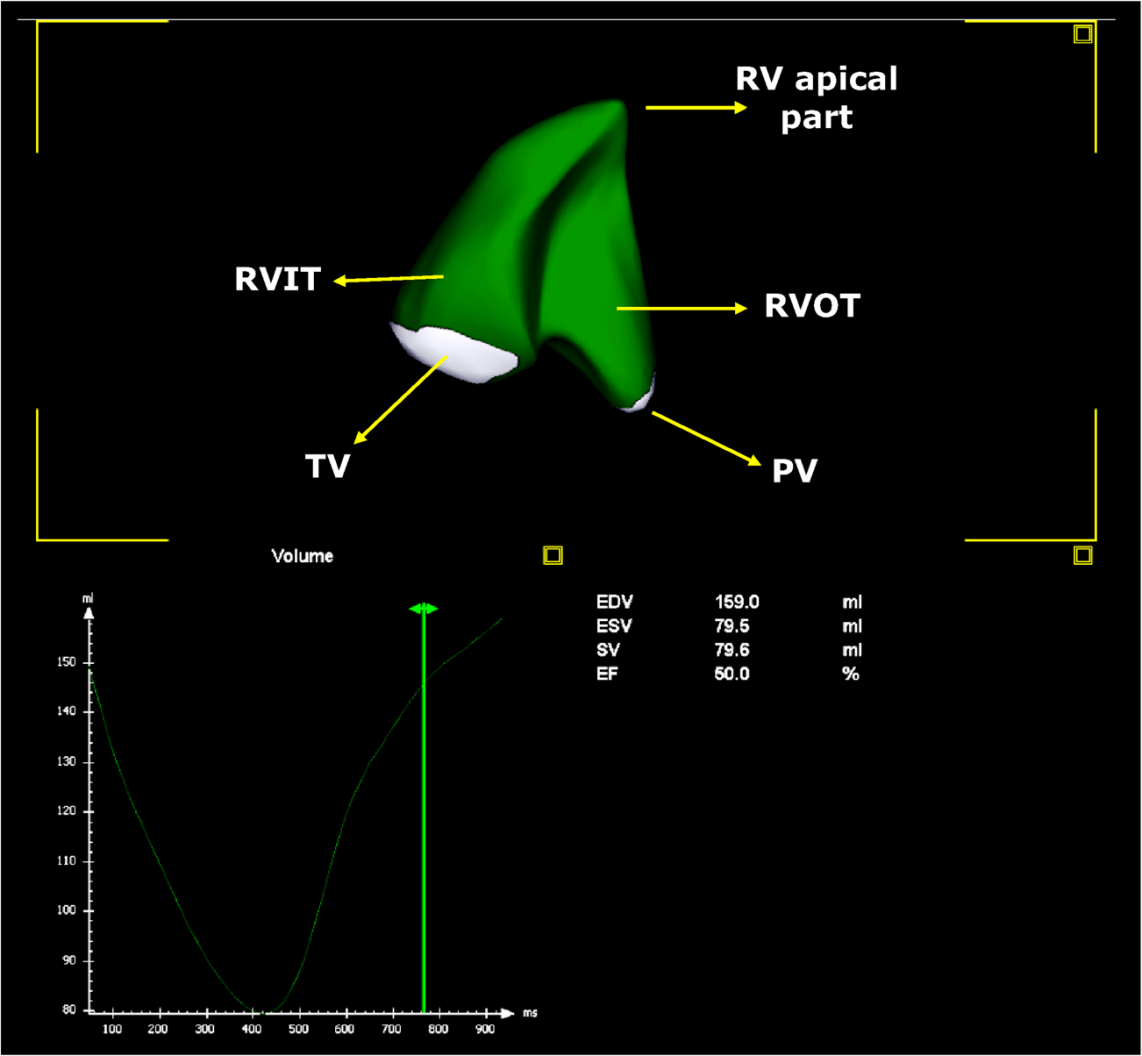
Right ventricular assessment by 3D-echocardiography. This picture shows a 3D-echocardiographic reconstruction of the right ventricle (RV). The software provides a motion picture of the RV as well as the volume-time curve and RV measures, such as RV end-diastolic volume (EDV), RV end-systolic volume (ESV), RV stroke volume (SV), and RV ejection fraction (EF)

Moreover, recent technological advances in echocardiography (such as 3DE and STE) have allowed, in patients with adequate acoustic windows, a more accurate evaluation of RV geometry, dimensions, and systolic function, both regional and global, thus overcoming many of the limitations and assumptions necessary for the standard 2D evaluation (Fig. 2) [20]. Two new such parameters of RV function, specifically *RV Tei index* and *free wall RV strain* by STE, have been independently correlated with mortality, whereas such correlations have not been shown for any RV diameter, TAPSE or RVFAC [19, 21]. Furthermore, these indices have shown a correlation with the pulmonary embolism severity index (PESI) and its simplified version (sPESI) [19]too, which point out their possible role in improving mortality risk estimation.

*Right ventricular mechanical dyssynchrony* (RV-MD) is a manifestation of regional wall motion abnormalities and is more likely to occur in acute cor pulmonale due to a sudden pressure overload. Its assessment may play a key role in the differential diagnosis of acute from chronic cases, and the standardization of RV-MD quantitative measurement performed by STE could be superior to visual inspection or other qualitative assessments [22, 23].

To this point, Vitarelli et al. demonstrated that in submassive APE, the impairment of RV segmental synchrony is associated with alterations of both right ventricular ejection fraction (RVEF), calculated by 3DE, and mid-free wall RV strain [24]. This result, together with those of the study mentioned before, suggests that RV strain analysis has a significant prognostic value, with an added reduction of inter-observer variability [25]. Furthermore, these studies have also demonstrated that once the RV overload has disappeared, the free wall RV strain returned to normal, while thr 3DE RVEF remained impaired during a 6-month follow-up [23]. Patients with APE presenting with *McConnell’s sign*, which consists of hypo-akinesia of the mid RV free wall with a normal function of the apex, show a lower absolute value of RV free wall apical segmental strain compared with controls [26]. This might be explained by the fact that the apical part of the RV free wall can be dragged by a hyper-dynamic LV, which makes it appear that this region has preserved contractility compared with the rest of RV free wall [26]. Interestingly, this specific alteration of apical free wall RV strain was more pronounced in APE than in other aetiologies of RV dysfunction, including idiopathic pulmonary hypertension (PH) [26].

Regarding the RA, evidence of its role in acute cor pulmonale is less consistent compared with the RV. In intermediate-risk APE patients with different morphological and functional alterations have been identified, such as an increased RA area, reduced RA strain by STE, and impaired RA/RV mechanical coupling relationship [27]. However, these findings have not been consistent across all studies, with another study showing no differences in RA size, both as area and volume, but only in RA strain between these patients and controls [28]. This result should stimulate future researches to validate the potential role of RA strain in the differential diagnosis of acute and chronic cor pulmonale. In addition, the same study identified a possible regional strain pattern specific to APE with the basal and mid segments of both RA and RV free wall as the regions most affected [28]. While a reduction of the initial extent of this impairment has been proposed over time, its persistence in the long-term could represent a predictor of worse outcome in these patients [22].

## Chronic cor pulmonale

**Fig. 3.**
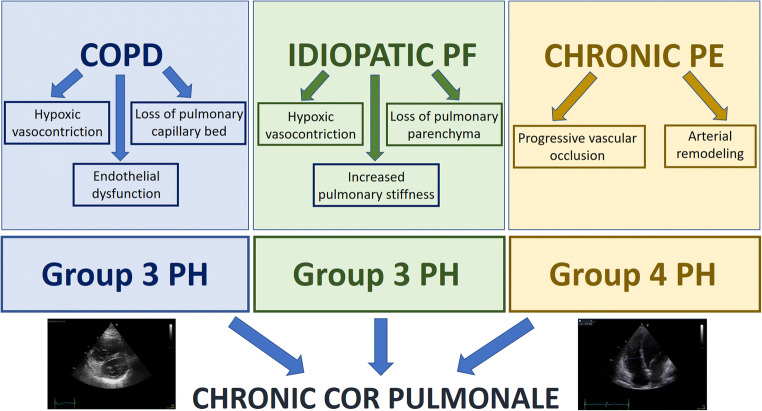
Main causes of chronic cor pulmonale. This figure shows the three most common disorders and their relative mechanisms, which are responsible for the development of chronic cor pulmonale: chronic obstructive pulmonary disease (COPD), idiopathic pulmonary fibrosis (PF), and chronic thromboembolic disease. PH, pulmonary hypertension; PE, pulmonary embolism

Chronic cor pulmonale refers to the clinical setting in which the RV has to face a progressively increasing afterload, which then determines its extensive dilatation and remodelling. Although the underlying causes may be different, PH is the common denominator of all the diseases that leads to chronic cor pulmonale. PH is defined as an increased mean pulmonary arterial pressure (PAP) ≥ 25 mmHg at rest, assessed by right heart catheterization [29]. Current guidelines classify idiopathic arterial PH in group 1, whereas PH due to pulmonary diseases and/or hypoxia is listed in group 3 and CTEPH in group 4 (Fig. 3) [30]. These are the most common conditions that are responsible for the development of chronic cor pulmonale, which by definition excludes RV dysfunction due to left heart diseases (group 2). In particular, the onset of PH as a complication of chronic lung diseases is a strong predictor of mortality [31]. Even though the diagnosis is based on the results of right heart catheterization, echocardiographic evaluation is a valuable tool, since it assesses the probability of PH in symptomatic patients, based on different parameters [30], which are detailed in Table 2.

**Fig. 4.**
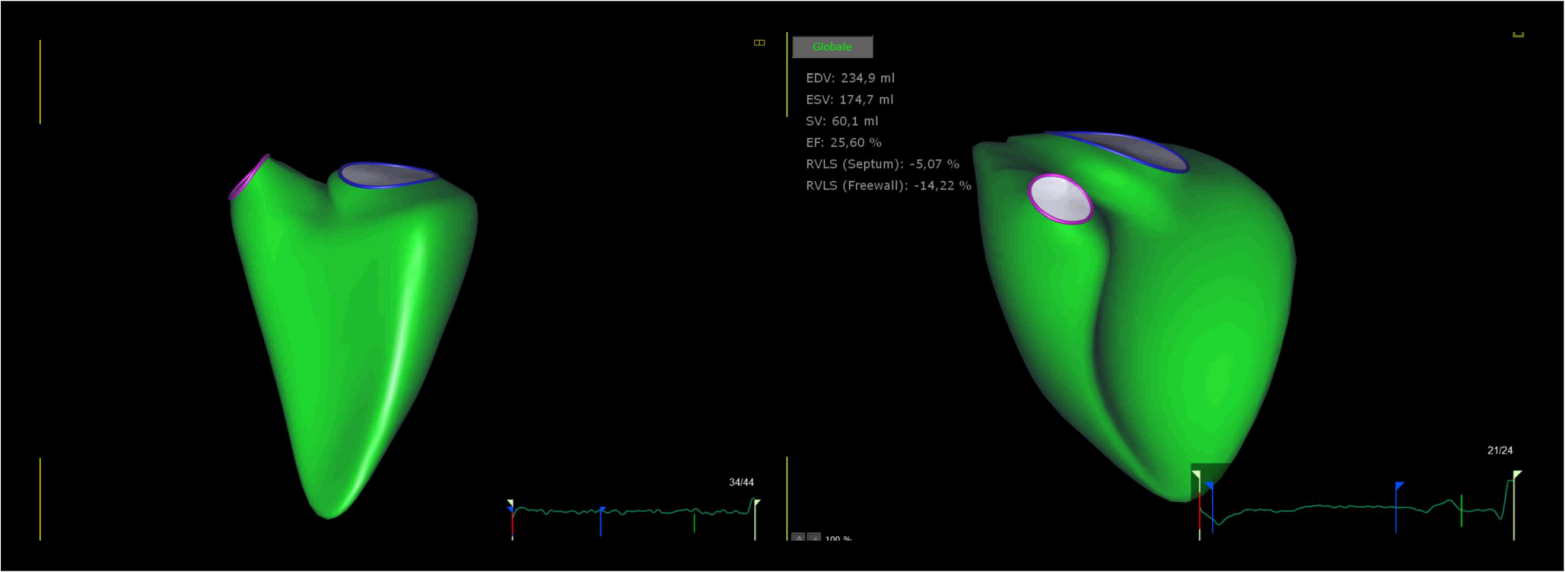
Morphological and functional difference between a healthy right ventricle and an impaired RV by 3D-echocardiography. The image on the left shows a healthy right ventricle (RV) that has a preserved triangular morphology, whereas the image on the right side shows a failing RV, with a markedly altered morphology and an impaired function, as indicated by the echocardiographic parameters listed in the picture. EDV, end-diastolic volume; EF, ejection fraction; ESV, end-systolic volume; RVLS, right ventricular longitudinal strain; SV, stroke volume

Several studies have demonstrated the prognostic role of echocardiography in the setting of PH, through parameters, such as TAPSE, RA size, the presence of pericardial effusion or RV longitudinal strain by STE [32, 33]. Specifically, a reduced STE RV longitudinal strain has been independently associated with a higher NYHA functional class and elevated N-terminal pro brain natriuretic peptide (NT-pro-BNP) levels [34], and, together with RV end-systolic diameters, has been found to be a strong predictor of outcome in these patients [34, 35]. Over the years, 3DE has become a valid alternative for RV quantification, since it overcomes some of the drawbacks of 2DE, such as foreshortening and geometrical assumptions that characterize the latter method. As a matter of fact, numerous studies have validated this technique against cardiac magnetic resonance measurements [36], especially RV volumes and ejection fraction, attesting also its additional prognostic value in PH patients [37]. For instance, in chronic PH, 3D, 2D-STE, and 3D-STE parameters are better indicators of global and regional RV dysfunction compared with traditional indices of RV [38]. In studies using 3DE in patient with PH, morphological RV changes (Fig. 4) have been shown to have a higher clinical utility over functional ones, with one study showing *RV end-systolic volume* as a stronger predictor of outcomes compared with *RV longitudinal strain* [35]. Similar to this finding, Amsallem et al. proposed a simple and reproducible 2D echocardiographic parameter called *RV end-systolic remodelling index*, defined as the ratio between end-systolic RV free wall and interventricular septum length, which was proven to have a strong prognostic value and could possibly help in the risk stratification of these patients [39]. Furthermore, considering the relationship between RV end-systolic dimensions and pressure, they defined three zones of adaptation: the first one is characterized by minimal RV dilatation with increasing RV pressure, the second one includes mixed modifications of adaptation and remodelling, whereas the third zone is defined by a progressively dilated RV with decreasing pressures as a result of lower cardiac output [39]. In this regard, we can consider the natural history of RV alterations in PH as undergoing two different types of remodelling: an “adaptive” one, with concentric RV hypertrophy and preserved systolic and diastolic function, and a “maladaptive” one, where the RV starts to fail presenting progressive enlargement and gradual functional decrease [40]. In addition, once the RV becomes dilated, the tricuspid annulus increases its diameter and tricuspid regurgitation develops, worsening RV functional capacity [41]. The detection of incipient RV morphology changes that are preludes to a failing heart is important, since the transition from the adaptive phenotype to the maladaptive one might occur even if the patient is in a stable clinical condition [42].

The role of cardiac fibrosis in developing overt RV failure in PH is not unequivocal, with both positive and negative features associated, as thoroughly analyzed by Andersen et al. [43]. In this regard, a possible dual role has emerged: at the beginning of the disease, it could represent an adaptive response, as opposed to later alterations in the collagen network of the interstitium, which might exert a detrimental role in the natural history of the disease. In particular, diffuse myocardial fibrosis contributes to RV diastolic impairment, which might represent an early marker of RV dysfunction, since it can occur even in the presence of preserved RV systolic function [44].

Another aspect to consider is that the RV dysfunction might be concealed at rest and become evident during exercise, highlighting the role of stress testing in the assessment of the RV. In fact, exercise echocardiography provides useful information on how hemodynamic changes during exercise in patients affected by or at risk of PH [45]. Indeed, several studies have demonstrated its additional prognostic value in patients with pulmonary arterial hypertension [46, 47]. In this context, the most commonly used “stressor” is exercise, since it represents a more physiological stimulus, even though hypoxia and dobutamine have also been employed [48]. In light of the valid information that could be gained from stress testing, newer techniques have been developed, such as exercise magnetic resonance [49].

The RA plays a key role in chronic cor pulmonale, since the augmented RV afterload induces a progressive RA dilatation, probably caused by impaired filling of the RV. Indeed, RA size has found to be a predictor of poor outcome in PH [50]. In addition to that, RA pressure reflects RV overload [51] and, together with PVR, is strongly correlated with RA total strain, which is significantly reduced as the functional capacity worsens, as assessed by the World Health Organization Functional Class (WHO-FC) [52]. In WHO-FC II and III, RA reservoir and conduit functions were found to be impaired, whereas RA active contractile function was preserved and responsible for a greater proportion of RV filling, unlike in WHO-FC IV where it also became impaired [52]. This might suggest a compensatory mechanism that takes place in the early stages of the disease, but once the preload reserve reaches its limit, it begins to fail. This concept is supported by the identification of an augmented RA emptying fraction in patients with mild-to-moderate PH, which decreases in severe PH, in line with the degradation of RA systolic function [53]. All these evidences suggest that RA strain might serve as a useful non-invasive parameter to evaluate the severity of PH.

### Chronic obstructive pulmonary disease

COPD is a chronic and progressively disabling disease, characterized by persistent respiratory symptoms and an irreversible clinical course, usually caused by significant exposure of the airways and/or alveolar department to noxious agents [54]. PH is a common complication in patients with severe or very severe COPD and is associated with significant morbidity and mortality. The development of PH in COPD is provoked by hypoxic vasoconstriction, loss of pulmonary capillary bed (in patients with emphysema), and endothelial dysfunction [55, 56]. Increasing evidence suggests that the structural and functional changes of the RV in COPD patients may significantly contribute to the diminished functional pulmonary activity, rather than attributing it only to airflow limitation and dynamic hyperinflation [57]. This confirms the importance of echocardiography in this setting, even though these patients can present a suboptimal acoustic window. In support of this hypothesis, E/A ratio of trans-tricuspid flow was found to be correlated with six-minute walk test (6MWT) distance, implying that RV diastolic function might contribute to patient exercise tolerance [57]. In the case of an inadequate tricuspid Doppler signal, it might be difficult to estimate the systolic PAP, hence other parameters might be used to overcome this problem, such as right ventricular systolic velocity (TDI s′ velocity) or pulmonary acceleration time adjusted for heart rate due to their correlation with mean PAP and PVR [58]. Another useful and promising index in this setting is free wall RV strain, which appears to be associated with elevated PVR, and is more feasible than the estimation of RV systolic PAP through tricuspid regurgitation velocity [59]. Furthermore, a recent study has demonstrated that patients with COPD present intra- and interventricular dyssynchrony, and the difference between the time-to-peak longitudinal systolic strain of the RV free wall and the one of the lateral wall of the LV is an independent predictor of rehospitalization within one year [60]. Regarding RA alterations in this disease, RA volume index has been associated with both systolic and diastolic dysfunction in PH due to COPD and has shown correlations with laboratory markers of heart failure and cardiac remodelling, such as NT-pro-BNP and ssT2 [61]. Moreover, as its value increases, so does the long-term mortality rate [62, 63].

### Idiopathic pulmonary fibrosis

IPF is the most common among the idiopathic interstitial pneumonias [64, 65] and is frequently complicated by PH, mostly in patients with advanced disease. The mechanisms leading to the development of PH associated to IPF are not fully understood. In addition to hypoxia, loss of pulmonary parenchyma and increased stiffness of the fibrotic lungs may contribute to the pathogenesis of PH-IPF [64, 66]. The most important determinant of survival in these patients is the RV capability to adapt to the elevated pulmonary vascular load [67]. Thus, echocardiography plays a central role both in initial assessment and during follow-up of these patients. As mentioned, RV global longitudinal strain is a strong predictor of outcome and functional capacity in PH, since it is also correlated with 6MWT distance, and the same is applied to PH due to IPF [68]. Furthermore, free wall RV strain, with a cut-off value of − 12%, was the only independent predictor of cardiovascular events during follow-up, in IPF, including sudden cardiac death [69]. However, it has been shown that RV functional impairment is more evident during exercise [70, 71], which highlights the role of stress echocardiography in assessing both RV contractile reserve and changes in pulmonary haemodynamics during exercise [45, 72–74]. D’Andrea et al. demonstrated in their study that both RV global and free wall longitudinal strain were impaired at rest, while TAPSE and s′ velocity were normal, showing that STE is particularly useful in assessing subclinical dysfunction [75]. In addition, a direct relationship between the reduction of RV function and impairment of exercise capacity has been proven in these patients [76]. Finally, free wall RV strain was significantly associated with diffusion lung carbon monoxide, which is a relevant predictor of 2-year mortality from the time of diagnosis [77]. This evidence suggests that RV strain might be useful in detecting early RV impairment and selecting patients that need to be more closely followed over time.

### Chronic thromboembolic pulmonary hypertension

CTEPH is characterized by a progressive occlusion of branches of pulmonary arteries by organized thrombi, which consequently leads to the onset of PH and is classified in group 4 according to current guidelines [30]. Regarding the role of echocardiography, recent evidence suggests that RV free wall strain can play an important role. It has shown good correlations with RVEF estimated by cardiac magnetic resonance imaging and a greater sensibility in detecting RV dysfunction compared with RVFAC, which is characterized by low reproducibility, using the cut-off value of − 20% [78]. There are still some conflicting data, with one study finding that RV basal free wall longitudinal strain impairment can be associated with an increased mean PAP [74] and another showing conflicting results with no correlation between RV global longitudinal strain and systolic PAP [78]. Evidence concerning the role of the RA in this clinical setting is not particularly consistent, and still some interesting findings emerged in a study that demonstrated both RA area and RV area decrease during treatment with Riociguat, which resulted in improvement of RV function and hypertrophy [79].

## The role of echocardiography in differential diagnosis between acute and chronic cor pulmonale

**Fig. 5.**
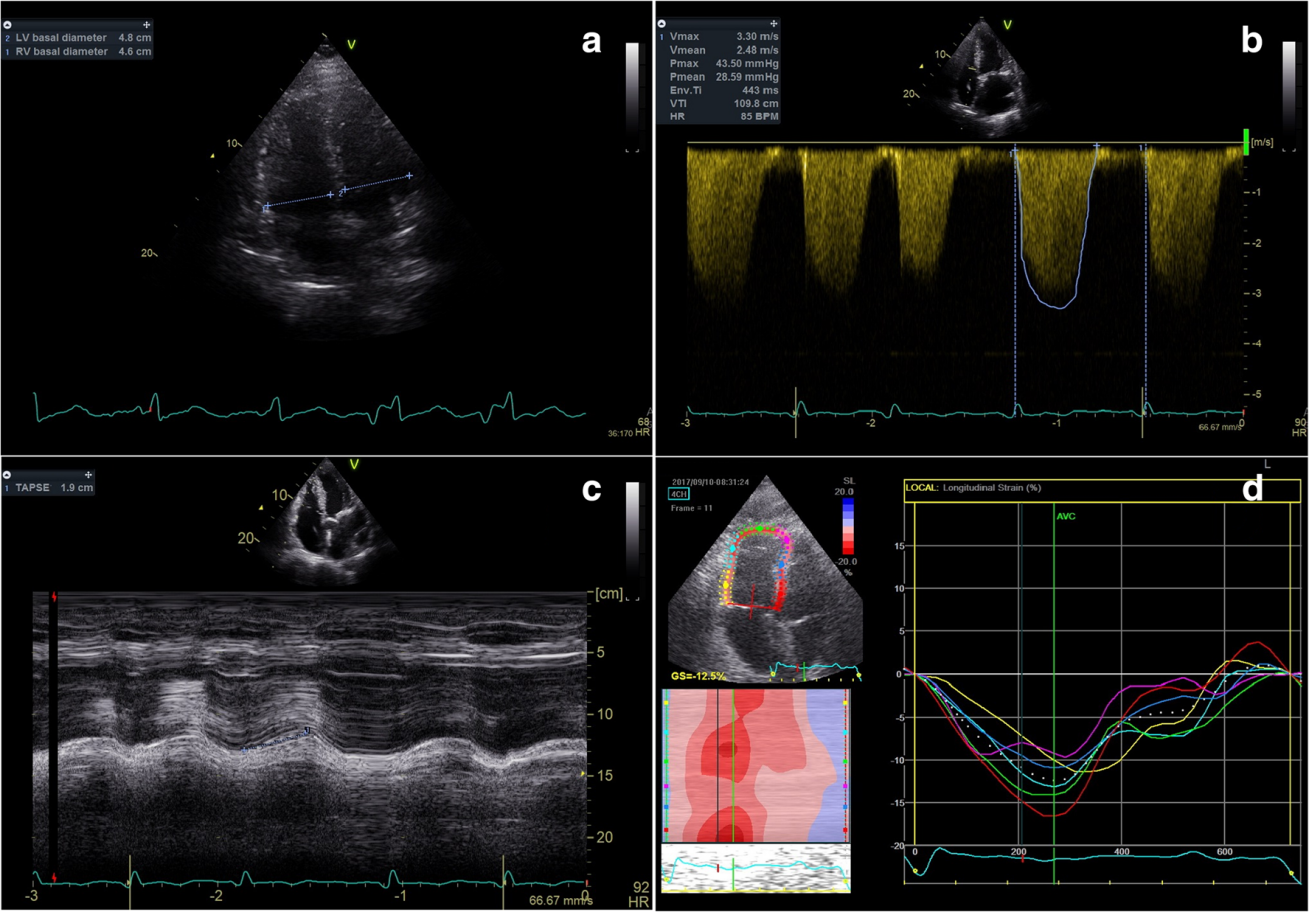
Relevant echocardiographic parameters found in acute pulmonary embolism. This figure shows the most common findings that can be found in the setting of acute pulmonary embolism. (**a**) Ratio between right ventricular (RV) and left ventricular (LV) basal diameters, often enough > 1, meaning a RV dilatation. (**b**) Right atrio-ventricular gradient, rarely above 60 mm Hg in the acute setting. (**c**) Tricuspid annular plane systolic excursion (TAPSE), which in acute setting is not necessarily reduced compared with controls.(**d**) Reduced RV longitudinal strain

**Fig. 6.**
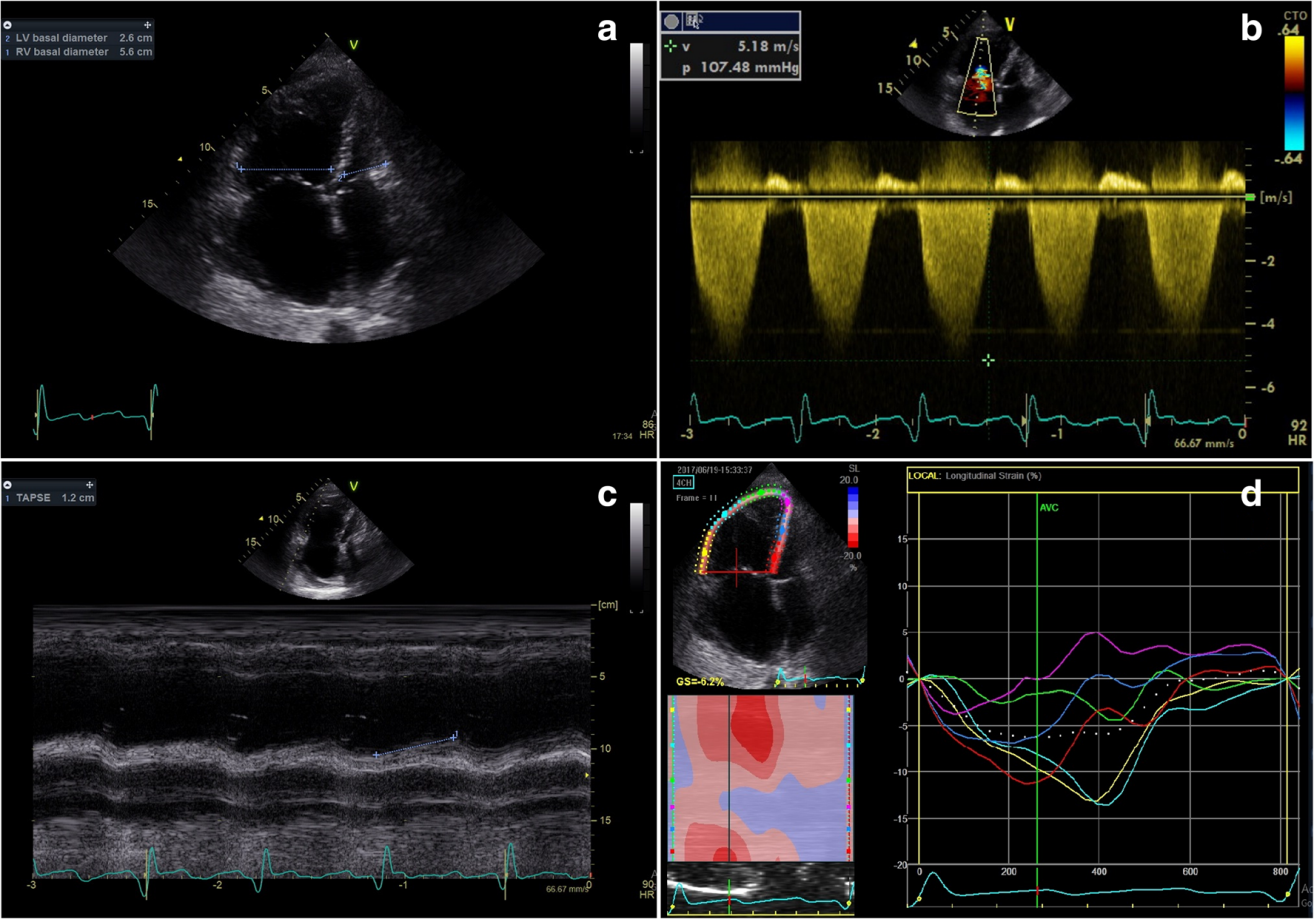
Relevant echocardiographic parameters found in chronic cor pulmonale. This figure shows the most common findings that can be found in the setting of chronic cor pulmonale. (**a**) Ratio between right ventricular (RV) and left ventricular (LV) basal diameters, often enough > 1, meaning a RV dilatation. (**b**) High right atrio-ventricular gradient, typical of chronic pulmonary hypertension. (**c**) Reduced tricuspid annular plane systolic excursion (TAPSE), reflecting a reduced RV longitudinal function. (**d**) Reduced RV longitudinal strain

The distinction between acute and chronic cor pulmonale is mainly based on patient history and clinical examination, although echocardiography always provides additional information and can sometimes play an important part. However, two conditions that might be hard to differentiate are APE and CTEPH. Whether echocardiography alone is able to distinguish one condition from the other is a difficult answer. In fact, current evidence is scarce and sometimes contradictory. Some studies that have tried to shed some light on this matter revealed that, both in the acute and chronic settings, the RV is affected by mechanical dyssynchrony [22, 23] that is more likely to happen with a rapid RV afterload increase. There are some specific patterns that can aid distinction, like in APE, where a more impaired function of the RV basal and mid free wall is found, while in CTEPH, there is advanced RV remodelling with marked hypertrophy. It is also important to underline that due to ventricular mechanical coupling, RV deterioration always influences LV performance [10]. Interestingly, the regional impairment of LV function might also depend on the timing during which RV pressure overload takes place, as was observed in the LV longitudinal strain analysis performed in different pulmonary diseases [10]. In the acute setting, LV global longitudinal strain is altered, caused mainly by regional peak systolic strain impairment of the septal, apical, and lateral segments, whereas in chronic pulmonary disease, longitudinal strain was found to be altered only in the septum, usually leading to a preserved LV global longitudinal strain [10]. Other RV functional parameters have been proposed that could help the distinction of the two scenarios, like right ventricular outflow tract (RVOT) systolic excursion, which is measured as the ratio between the difference of RVOT end-diastolic diameter and RVOT end-systolic diameter divided by the RVOT end-diastolic diameter [80]. This parameter was found to have lower values in patients presenting with APE, as opposed to those with chronic pulmonary diseases [80]. One must also consider that when estimating systolic PAP through tricuspid regurgitation, the RV cannot generate pressures greater than 40–50 mmHg in order to overcome an acute afterload increase [81]; therefore, the presence of a higher tricuspid regurgitation pressure gradient should arouse suspicion of a pre-existing PH. Figures 5 and 6 show common echocardiographic findings in APE and CTEPH, respectively. Although, to date, there is no conclusive evidence of any echocardiographic parameter that could on its own reliably guide the differentiation of acute and chronic cor pulmonale, this gap in knowledge should be viewed as an opportunity for further research on this topic.

## The role of echocardiography during follow-up

In the context of both acute and chronic cor pulmonale, it is essential to stress that echocardiography fulfils a role that goes beyond a diagnostic and prognostic purpose. Indeed, the echocardiographic exam could help the clinician to identify the patients that could benefit from a particular treatment and most importantly could attest the success of the adopted therapeutic strategy. In the acute setting, the reduction of RV size and its recovery, as well as the reduction of systolic PAP, are parameters that could indicate that the anticoagulant regimen has been effective. Moreover, after the acute episode, as the current guidelines recommend [4], it is important to pursuit a regular echocardiographic follow-up, as a screening for PH due to chronic pulmonary embolism. In the context of chronic cor pulmonale, especially regarding pulmonary arterial hypertension, the echocardiographic evaluation plays a central role in the sequential follow-up that these patients should undergo [30]. In fact, systolic PAP is often used as a surrogate for invasive haemodynamic assessment, even though systolic PAP, RV function, and RV size do not necessarily follow the same path [82]. In fact, RV function might become impaired during follow-up, in spite of little increments in systolic PAP. Wright et al. have evaluated the relative value of baseline and follow-up echocardiographic evaluation of both RV function and systolic PAP in patients with pulmonary arterial hypertension receiving vasodilator therapy [83]. They found that changes during the therapeutic regimen were minimal and only modifications in RV free-wall strain, inferior vena cava, and RA area were prognostic predictors in this population [83].

## Cor pulmonale parvus: a new entity?

The MESA COPD study defined a new RV phenotype in COPD patients, termed “cor pulmonale parvus,” defined by a lower RV volume, normal RV mass or RVEF, but with increasing functional impairment, as assessed by cardiac magnetic resonance imaging [84]. Another interesting finding of the study was the fact that both RV end-diastolic volume and stroke volume progressively decreased as the severity of emphysema increased, particularly in those with centrilobular and paraseptal emphysema [84]. These results might appear in contrast to the common cardiac RV phenotype in COPD patients, characterized by progressive RV hypertrophy, dilatation, and ultimately pump dysfunction, as mentioned earlier. Although the results seem interesting, the pathophysiology is still not completely clear, as Reichek pointed out in his accompanying editorial [85]. One possible explanation for the smaller RV size is the anomalous position of the diaphragm that might compromise the inferior vena cava, or it could be a result of RV diastolic dysfunction and increased intrathoracic pressure that leads to a reduced venous return to the thorax [84]. On the other hand, it might be the expression of RV concentric remodelling in response to mild increases in PAP, but more research is needed on this RV phenotype.

## Conclusions

Echocardiography plays a central role in the primary assessment of RV morphology and function in all forms of PH, providing an accurate insight into pathophysiological changes and carrying an important diagnostic and prognostic value.

This review pointed out how several functional and morphological echocardiographic RV parameters, especially those derived from new techniques, represent valuable and easily accessible tools for the clinician in order to assess RV function in patients with cor pulmonale and periodically reassess functional stability, deterioration or improvement over time.

Regarding future perspectives, further studies are required to investigate how new echocardiographic parameters could improve discrimination between acute and chronic cor pulmonale, which would be particularly useful in defining the optimal therapeutic strategy. Furthermore, it might be intriguing to establish the role of echocardiography in COPD patients, whether it is in aid of identifying patients with a proportionally greater lung destruction or an inflammation of the airways, or investigating the new phenotype “cor pulmonale parvus.”
